# Bioelectrical impedance analysis and skinfold thickness for the estimation of body fat: a population-based study in southern Brazil

**DOI:** 10.1590/1806-9282.20240406

**Published:** 2025-03-17

**Authors:** Clovis Arlindo de Sousa, Bruna de Macedo, Luciane Coutinho de Azevedo, Nagila Raquel Teixeira Damasceno, Till Ittermann, Henry Völzke, Marcello Ricardo Paulista Markus, Ernani Tiaraju de Santa Helena

**Affiliations:** 1 University of Blumenau, Department of Health Sciences, Postgraduate Program in Collective Health – Blumenau (SC), Brazil.; 2 Istituti Clinici Zucchi Monza, Department of Emergency – Monza, Italy.; 3 University of São Paulo, Heart Institute (InCor) of the Medical School Hospital – São Paulo (SP), Brazil.; 4 University Medicine Greifswald, Institute for Community Medicine – Greifswald, Germany.; 5 University Medicine Greifswald, Institute for Community Medicine, German Centre for Cardiovascular Research (DZHK), Partner Site Greifswald – Greifswald, Germany.; 6 University Medicine Greifswald, German Centre for Cardiovascular Research (DZHK), German Center for Diabetes Research (DZD), Department of Internal Medicine, Partner Site Greifswald – Greifswald, Germany.

**Keywords:** Body composition, Bioelectrical impedance, Skinfold thickness, Body fat distribution, Cross-sectional study, Epidemiology

## Abstract

**OBJECTIVE:**

The objective of this study was to compare bioelectrical impedance analysis and skinfold measurements for the estimation of body fat in adults.

**METHODS:**

We analyzed data from 292 adult participants enrolled in a cross-sectional population-based study. Four skinfold measurements were performed, and body fat percentage was estimated using the Petroski formula. Bioelectrical impedance analysis was performed using a tetrapolar electrical bioimpedance device. The measurements were compared using Student's t-test, Robinson's coefficient of agreement, Cronbach's alpha, and linear regression models (slope and intercept).

**RESULTS:**

The mean percentage of body fat estimated by skinfold measurements was higher compared to bioelectrical impedance analysis (29.0 vs. 27.9; p<0.001), but the agreement between the methods is good (alpha=0.88; Robinson's coefficient of agreement=0.91). Linear regression models showed a good correlation (r^2^=0.69). Bland-Altman analysis showed a mean difference of −1.02 (−1.54 vs. −0.50) between the two techniques. The agreement was better in women, those aged 20-39 years, those with a body mass index<25, and those with a waist-to-height ratio<50.

**CONCLUSION:**

The two methods showed a good agreement between the mean values of body fat percentage and can be used in population studies. However, their results should be considered with caution in men, people aged 40 years and older, overweight people, and those with a waist-to-height ratio≥50.

## INTRODUCTION

The search for accurate methods of estimating body composition is a constant concern of the medical and scientific community, with the aim of obtaining information that will allow a consistent diagnosis of the nutritional status of individuals and populations^1,2^.

Hydrostatic weighing, densitometry, air displacement plethysmography, and magnetic resonance imaging are considered gold standard methods for estimating body fat^3^. However, the use of these methods in epidemiological studies is limited due to the technical qualification required to perform them, the time required, and the high cost^2,3^.

Bioelectrical impedance analysis (BIA) has been used in clinical practice and epidemiological studies because it is a non-invasive method and provides rapid estimates of body composition^3,4^. However, its findings may be influenced by hydration status, hydroelectrolyte balance, and some clinical conditions of the individual, and may vary between ethnic groups^5^.

Skinfold measurements provide a simple estimate of body composition by assessing the thickness of the subcutaneous fat in millimeters. Previously validated formulae are applied on these measurements to estimate the percentage of body fat^6,7^. The accuracy of skinfold measurements depends on the technical quality of the measures taken and on using appropriate formulae to the population studied^8^.

Studies have shown a good correlation between BIA and skinfold methods for estimating fat mass^9,10^. However, these studies were conducted in clinical settings^11,12^ and in specific populations such as young adults^13^, children and adolescents^14^, elderly^15^, and military personnel^16^ and/or have small sample sizes. Therefore, studies comparing these two methods in population-based epidemiological studies are needed.

Hence, this study aimed to compare the BIA and skinfold measurement methods for estimating body fat in adults enrolled in a population-based study in southern Brazil.

## METHODS

### Study design

Data were derived from the baseline of the cohort "Study of Health in Pomerode (SHIP-Brazil)" described previously^17^. In short, a stratified sample of 2,488 individuals of both sexes and aged between 20 and 79 years, who had lived in Pomerode (SC) for at least 6 months, were included in the study. Participants were assessed by trained and certified staff in the University Hospital of Blumenau. The data collection procedures were described in the standard operating procedures, available at www.furb.br/vspomerode. All participants signed a written informed consent. The SHIP-Brazil was approved by the Research Ethics Board of the University of Blumenau (CAAE: 99559118.0.0000.5370) and complied with the Declaration of Helsinki.

Of the 1,755 SHIP-Brazil adult participants (20 and 59 years), 1,490 (85%) had weight and height measurements, 1,484 (84.6%) had waist measurements, 1,035 had skinfold measurements, and 448 had BIA testing. Finally, 292 participants had all measurements and were included in this study. There were no statistically significant differences between the participants included in the study and those who did not have all the measurements available for the variables studied.

### Anthropometric measurements

Body mass (in kilograms) was measured using a W300 electronic scale (WELMY^®^, Brazil). Participants stepped onto the center of the platform barefoot, wearing minimal clothing and no jewelry. Height (in centimeters) was measured using a stadiometer with a rod attached to the scale. Body mass index (BMI) was calculated by dividing the body mass (kg) by the square of height (meters).

Waist circumference was measured with an inelastic tape measure (Cescorf^®^, Brazil) graduated in centimeters. The tape measure was positioned in the abdominal region at the medial point between the last rib and the right superior iliac crest. The waist-to-height ratio (WtHR) was calculated by dividing the waist circumference by the height (centimeters). Participants with a WtHR<0.50 were considered to be at low cardiometabolic risk^18^.

Skinfolds were measured with a scientific adipometer (Cescorf^®^) graduated in tenths of millimeters. The skinfold measurements were taken parallel to the longitudinal axis of the measured segment and in the standing position. The triceps and the subscapular, suprailiac, and medial calf folds were measured.

### Body fat percentage

We adopted the body density formulae proposed by Petrosky^6,7^ to estimate body fat from skinfolds since they have been validated in a sample of adults from southern Brazil, namely:

1. Men (18-66 years old): 1.10726863—(0.00081201*X4)+(0.00000212*X4^2^)—(0.00041761*age);
2. Women (18-51 years old): 1.02902361—(0.00067159*X4)+(0.00000242*X4^2^)—(0.00026073*age)—(0.00056009*weight)+(0.00054649*height), where X4=sum of the subscapular, triceps, suprailiac, and calf folds.

The Siri equation^19^ was used to convert body density into body fat percentage.

A tetrapolar bioimpedance device (Bodystat 1500MDD, UK) was used to estimate the percentage of body fat. All possible factors that could interfere with the results were checked before the examination. The participants were placed in the supine position with the legs and arms away from the body, without contact with any metal objects. The skin of the limbs was cleaned, and electrodes were placed and connected to the cables of the BIA device.

### Statistical analysis

The Bland-Altman plot was used to analyze the residuals between BIA body fat percentage and skinfold body fat percentage measurements. This approach was further stratified by sex, age group, and BMI.

Student's t-test was used to compare means. Cronbach's alpha test was used to estimate the internal consistency of each measure, and RAC was used to estimate the correlation between the BIA body fat percentage and skinfold body fat percentage (perfect agreement=1). Linear regression models were fitted between the BIA body fat percentage and skinfold body fat percentage crude and stratified for sex, age group, BMI, and WtHR. A perfect agreement was defined as a regression line slope of 1.0 and a regression line intercept at zero. Differences in slope from 1.0 and intercept from zero were tested for significance using a Student's t-test. A p<5% was considered statistically significant.

## RESULTS

The mean age of the participants was 41.7 years (dp=9.9), and 162 (55.5%) of them were male. Men were older (44.2 vs. 38.7; p<0.001), with a higher BMI (28.9 vs. 27.4; p<0.001) and a higher WtHR (54.7 vs. 50.7; p<0.001).

**Figure 1:**
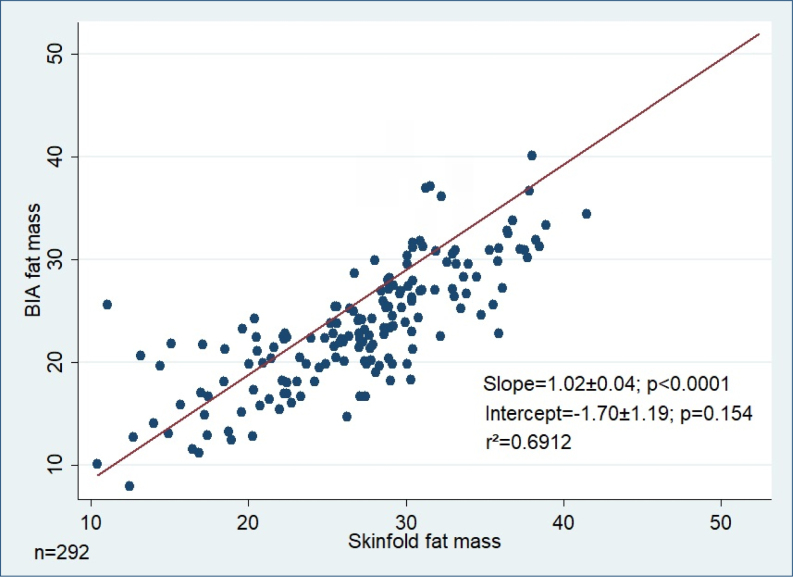
A linear regression model comparing bioelectrical impedance analysis fat mass and skinfold fat mass. BIA: bioelectrical impedance analysis.

**Figure 2:**
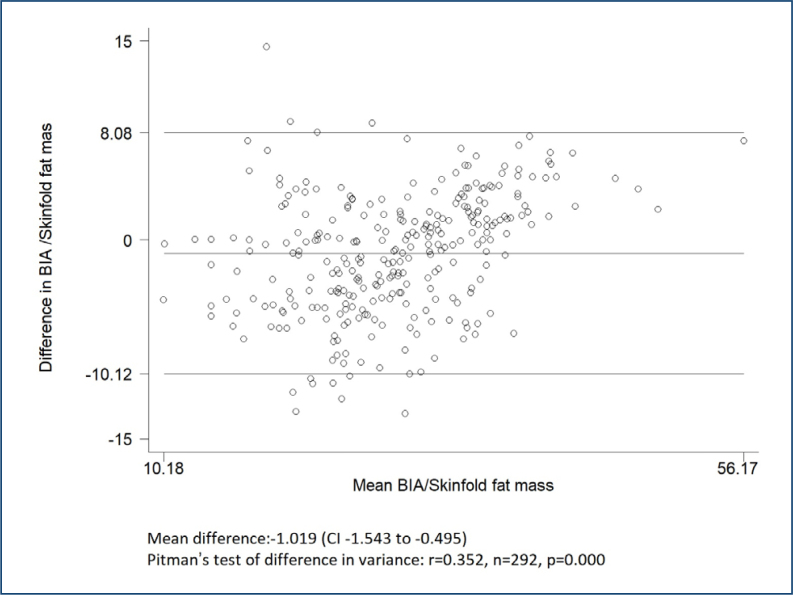
Bland-Altman plot of differences between bioelectrical impedance analysis fat mass and skinfold fat mass. BIA: bioelectrical impedance analysis; CI: confidence interval.

The linear regression model of mean skinfold and BIA body fat percentages showed a good correlation with r^2^=0.69. There was a significant deviation from the line of unity (1.02±0.04, p=0.001). The intercept did not differ from zero (−1.70±1.19) (p=0.154). The Bland-Altman analysis revealed a mean difference of −1.02 (−1.54 vs. −0.50) between the two techniques. Figures 1 and 2 show the agreement analysis between the methods using linear regression models and the Bland-Altman plots.

The mean body fat percentage was lower when estimated by BIA (27.9 vs. 29.0, p<0.001). The internal consistency (alpha) between the measurements was =0.8978. The RAC was 0.9072. The full comparison of mean skinfold and BIA body fat percentages, their RAC, and internal consistency by study variables is shown in Table 1.

**Table 1:** Comparison of body fat percentages by bioelectrical impedance analysis and skinfold measurements, Robinson's coefficient of agreement, and Cronbach's alpha for the study variables, Study of Health in Pomerode-Brazil, 2014-2018 (n=292).

Variables		%BF-BIA Mean (SD)	%BF-Sk Mean (SD)	p	RAC	A
Sex						
	Male (n=162)	23.4 (6.0)	26.9 (6.9)	<0.001	0.8925	0.8795
	Female (n=130)	33.7 (6.9)	31.6 (6.1)	<0.001	0.9506	0.9480
Age group (years)						
	20-39 (n=119)	26.7 (8.5)	27.4 (6.5)	0.093	0.9083	0.8991
	40-59 (n=173)	28.9 (7.9)	30.1 (6.6)	<0.001	0.9026	0.8921
BMI (kg/m^2^)						
	Less than 25 (n=85)	23.4 (6.8)	23.4 (5.8)	0.999	0.8600	0.8373
	25 and above (n=207)	29.8 (8.0)	31.3 (5.7)	<0.001	0.8968	0.8849
WtHR						
	Low risk (n=110)	24.9 (7.7)	24.8 (6.3)	0.716	0.9235	0.8937
	High risk (n=182)	29.8 (7.9)	31.5 (5.5)	<0.001	0.8764	0.8767

BMI: body mass index; SD: standard deviation; %BF-BIA: body fat percentage by tetrapolar bioimpedance; %BF-Sk: body fat percentage by skinfold; t: Student's t-test; p: p-value; RAC: Robinson's coefficient of agreement; α: Cronbach's alpha; BIA: bioelectrical impedance analysis; SHIP: Study of Health in Pomerode; WtHR: waist-to-height ratio.

The analysis of agreement between BIA and skinfold methods stratified by sex, age group, BMI, and WtHR revealed better agreement in women (mean difference=2.104, 95% confidence interval [CI] 1.602; 2.607), those aged 20-39 (mean difference=-0.708, 95%CI −1.536; 0.119), those with a BMI<25 (mean difference=0.001, 95%CI −1.001; 1.002), and those with a WtHR<50 (mean difference=0.154, 95%CI −0.665; 0.973).

## DISCUSSION

In our study, the mean value of the skinfold-based calculation of body fat percentage was higher than that estimated by BIA. The mean differences between the two measurements ranged from 1.2 to 3.5% points. Participants aged between 20 and 39 years, with a BMI of up to 25 kg/m^2^, with a high cardiovascular risk as estimated by the waist circumference and WHtR, and with a low cardiovascular risk had similar mean values for both skinfold and BIA body fat percentages. These methods showed better agreement and good internal consistency in the mean values of body fat percentages. Better agreement was observed in the following subgroups: women, participants aged 20-39 years, participants with a BMI<25, and participants with a WtHR<50.

The methods evaluated in this study showed better agreement (RAC=0.9072; alpha=0.898; r^2^ adjusted=0.69) than other studies with similar age groups or sample sizes (r^2^=0.630 and =0.525, respectively)^10,20^. However, our results were similar to those of previous studies with other populations, such as adult women (r^2^=0.900)^9^ and people older than 18 years with kidney disease (r^2^=0.860)^11^. In this study, age below 40 years showed the best agreement values between methods, perhaps because there is greater accuracy in younger people and also in women. Regression analysis showed that skinfold-based body fat percentage was strongly correlated with BIA-based body fat percentage. This result was consistent with that of others in different contexts^10,11^.

BIA has been considered a good alternative method for estimating the percentage of body fat because of its strong agreement with dual-energy X-ray absorptiometry (DEXA); however, individual characteristics must be analyzed, such as obesity level and race/ethnicity^1^. In obese individuals, BIA showed a variable correlation with DEXA in estimating the percentage of body fat^21^. DEXA and BIA methods may be interchangeable at the population level, but at the individual level, different BMI values may reduce the agreement between them^21^.

Skinfold measurements have also shown good accuracy and correlation with gold standard methods in adults^20^. Given the high concordance with BIA found here, the skinfold measurement method may be more viable for use in public health services with limited financial resources. Despite these positive aspects, the validity of skinfold measurements is highly dependent on the training of the examiners, the formula used, the number of skinfolds measured, and the degree of obesity present, as excess subcutaneous fat may prevent or limit a correct skinfold measurement^22^.

In this study, agreement is lower at the extremes of older age, higher BMI, and higher WHtR, possibly due to the greater operational difficulty of skinfold measurements of body fat in these groups. The mean values of body fat percentage using skinfold and BIA were different in individuals with a BMI≥25 kg/m^2^ (p<0.001) and in those with an adequate and high WHtR (p<0.001), although they still showed good internal consistency, with Cronbach's alpha of 0.885 and 0.877, respectively.

These results underline the importance of careful training of examiners and supervision while measuring skinfolds, especially in older people and those who are overweight or obese. Furthermore, the two methods studied estimate the percentage of total body fat but not visceral and subcutaneous fat. In this sense, the use of combined measurements such as abdominal circumference, waist-to-hip ratio, or WHtR is recommended in clinical and research practice for a more accurate assessment of metabolic and cardiovascular risks.

A potential strength of this study is that the body fat percentage estimated by BIA and skinfold measurements was compared in a population-based study of adults in southern Brazil. Although both methods seem feasible, the results should be treated with caution due to differences in BIA device types, skinfold measurement protocols, sample sizes, and target populations.

A potential limitation of the study was the lack of use of gold standard methods such as DEXA, which did not allow a direct comparison between the two methods used here. However, tetrapolar BIA can be considered an accurate and precise method for estimating body fat percentage.

## CONCLUSION

Our results indicate a good agreement between the mean values of the skinfold and tetrapolar BIA methods for estimating the body fat percentage in a population-based study of adults in southern Brazil. The highest agreement between the methods was found in younger individuals, women, individuals with a low WtHR risk, and eutrophic adults.

## Data Availability

The authors confirm that most of the data used in this article can be found in Table 1 and Figures 1 and 2. Any additional data are available on request.
